# Optimization of Non-Alloyed Backside Ohmic Contacts to N-Face GaN for Fully Vertical GaN-on-Silicon-Based Power Devices

**DOI:** 10.3390/mi15091157

**Published:** 2024-09-15

**Authors:** Youssef Hamdaoui, Sofie S. T. Vandenbroucke, Sondre Michler, Katir Ziouche, Matthias M. Minjauw, Christophe Detavernier, Farid Medjdoub

**Affiliations:** 1 Institute of Electronics, Microelectronics and Nanotechnology, CNRS-IEMN, 59650 Lille, France; katir.ziouche@univ-lille.fr (K.Z.); farid.medjdoub@univ-lille.fr (F.M.); 2 Department of Solid State Sciences, CoCooN Group, Ghent University, Krijgslaan 281/S1, B-9000 Ghent, Belgiummatthias.minjauw@ugent.be (M.M.M.); christophe.detavernier@ugent.be (C.D.); 3 Department Innovation Management Siltronic AG, Einsteinstraße 172, 81677 München, Germany; sondre.michler@siltronic.com

**Keywords:** GaN-on-Si, N-face N-GaN ohmic contact, backside contact, fully vertical, power devices

## Abstract

In the framework of fully vertical GaN-on-Silicon device technology development, we report on the optimization of non-alloyed ohmic contacts on the N-polar n+-doped GaN face backside layer. This evaluation is made possible by using patterned TLMs (Transmission Line Model) through direct laser writing lithography after locally removing the substrate and buffer layers in order to access the n+-doped backside layer. As deposited non-alloyed metal stack on top of N-polar orientation GaN layer after buffer layers removal results in poor ohmic contact quality. To significantly reduce the related specific contact resistance, an HCl treatment is applied prior to metallization under various time and temperature conditions. A 3 min HCl treatment at 70 °C is found to be the optimum condition to achieve thermally stable high ohmic contact quality. To further understand the impact of the wet treatment, SEM (Scanning Electron Microscopy) and XPS (X-ray Photoelectron Spectroscopy) analyses were performed. XPS revealed a decrease in Ga-O concentration after applying the treatment, reflecting the higher oxidation susceptibility of the N-polar face compared to the Ga-polar face, which was used as a reference. SEM images of the treated samples show the formation of pyramids on the N-face after HCl treatment, suggesting specific wet etching planes of the GaN crystal from the N-face. The size of the pyramids is time-dependent; thus, increasing the treatment duration results in larger pyramids, which explains the degradation of ohmic contact quality after prolonged high-temperature HCl treatment.

## 1. Introduction

**Figure 1:**
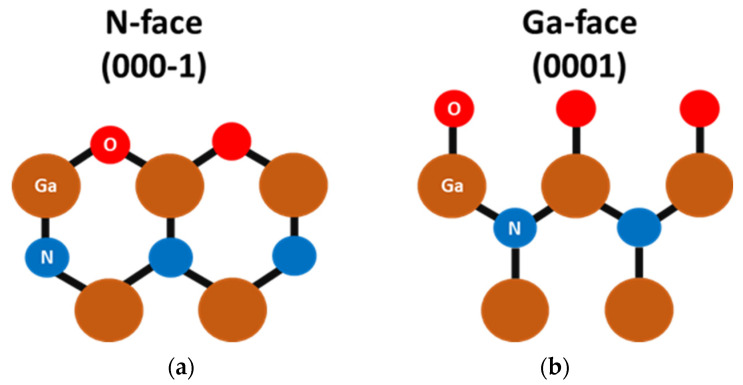
Side view diagram of the N-face (**a**) and Ga-face (**b**) GaN in the presence of oxygen atoms.

Gallium nitride (GaN)-based material has shown outstanding potential as a candidate for future power devices overcoming Silicon (Si) and Silicon carbide (SiC) limitations such as physical properties or cost of fabrication. Indeed, high GaN epi-layer quality can be grown on Silicon substrate, benefiting from high electron mobility, a high electric field, and a large band gap at Silicon cost. However, reliability issues still plague well-established lateral GaN high-electron-mobility transistors (HEMTs) [1,2]. Furthermore, unlike SiC-based devices, larger device dimensions and no avalanche soft breakdown can be achieved, which slows down their market penetration for medium- and high-voltage (HV) applications (beyond 600 V voltage operation). Vertical GaN technology grown on low-cost foreign substrates is a promising solution for medium- and high-voltage applications. This technology relies on junctions to provide high and reliable performance while potentially allowing a low cost of fabrication [3,4,5,6,7,8]. In this frame, GaN P-N junction-based vertical structures offer robust performance including avalanche breakdown capability and high current spreading while maintaining small device dimensions [3,9,10]. To maintain a low cost of fabrication, these devices can be grown on either Silicon or Sapphire substrate. In this case, most of the reported vertical GaN devices have a pseudo-vertical structure, where the backside layer is reached from the front side by dry etching. This structure is widely used since the process is relatively straightforward, as no backside processing is required to fabricate the discrete devices. On the other hand, unlike fully vertical structures, pseudo-vertical devices suffer from the so-called current crowding effect directly affecting the on-state behavior due to thermal dissipation issues [11]. It can be pointed out that this undesired effect scales with the device dimensions, increasing significantly the on-state resistance, which makes the fully vertical structure mandatory. The development of a fully vertical architecture based on GaN grown on Silicon or Sapphire substrate is challenging. Unlike Sapphire substrate, which implies the use of laser bonding [12] to obtain access to the backside n+ layer, the development of fully vertical GaN devices on Silicon substrate is achievable by means of a local substrate and buffer layer removal [13]. In this case, several challenges needed to be addressed including an optimized removal of Si and buffer layers without damaging the active device layers with a precise etching control to preserve the n+ layer thickness and the optimization of the backside ohmic contact by means of transmission line measurement. Indeed, one of the key challenges is the development of the backside ohmic contact on the n-face polarity of the n+-doped GaN layer. This change in the crystal polarity of GaN alters the physical properties of the material and influences device performance. Unlike the Ga-face (Figure 1b), the formation of low resistive ohmic contacts on the nitrogen face of the n+ layer is more complex due to the inversed polarity from the backside; the atom position in Wurtzite crystal of GaN is prone to high oxidation (Figure 1a) [14]. This problem is faced with the vertical GaN-on-GaN structure in the field of power electronics and optoelectronics [15,16,17,18,19,20,21,22,23] and also in the fully vertical GaN-on-Sapphire case [12]. However, this parameter has not been assessed on Silicon substrate due to the non-planar surface of the membrane, limiting the use of the standard lithography method. In this work, TLM lift-off inside GaN-on-Silicon membranes using laser direct lithography has been developed allowing proper characterization of the specific contact resistance on top of the n-face n+-doped GaN backside layer. The electrical results are supported by X-ray photoelectron spectroscopy (XPS) and scanning electron microscopy (SEM) analysis to reveal the impact of the wet treatment. Then, we propose a simple approach to overcome the limitation of the n-face n-GaN ohmic contact. A high-temperature HCl-based wet treatment of the GaN surface was developed, which significantly enhanced the ohmic contact quality under specific treatment conditions.

## 2. Device Fabrication and Characterization

A GaN-based PIN diode heterostructure was grown on a 6-inch Silicon substrate using metal-organic chemical vapor deposition (MOCVD). The GaN epitaxy started with the growth of a thick buffer layer, followed by an 800 nm n+-doped GaN layer with a Si-doping concentration of 5 × 10^18^ cm^−^^3^. Subsequently, a 3.5 µm n-doped GaN drift layer was grown with a Si-doping concentration of 3 × 10^16^ cm^−3^, corresponding to a net doping of 9 × 10^15^ cm^−^^3^ considering the residual carbon concentration. The growth was completed with a p-type GaN layer on top, doped with 2 × 10^19^ cm^−^^3^ of Magnesium (Mg), resulting in a hole concentration of 3 × 10^17^ cm^−^^3^ as assessed by Hall effect measurements.

**Figure 2:**
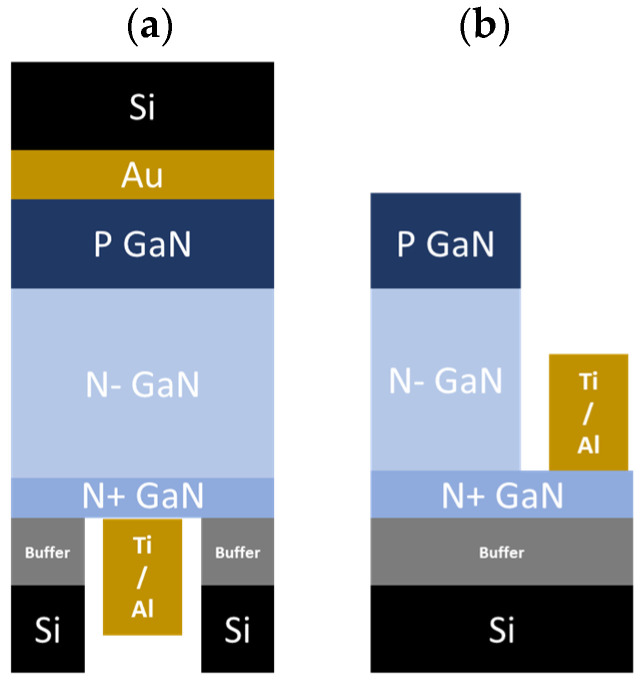
Schematic overview of the studied samples to characterize the ohmic contacts on N-face GaN layer, samples B–D (**a**), and Ga-face GaN layer, sample A (**b**).

**Figure 3:**
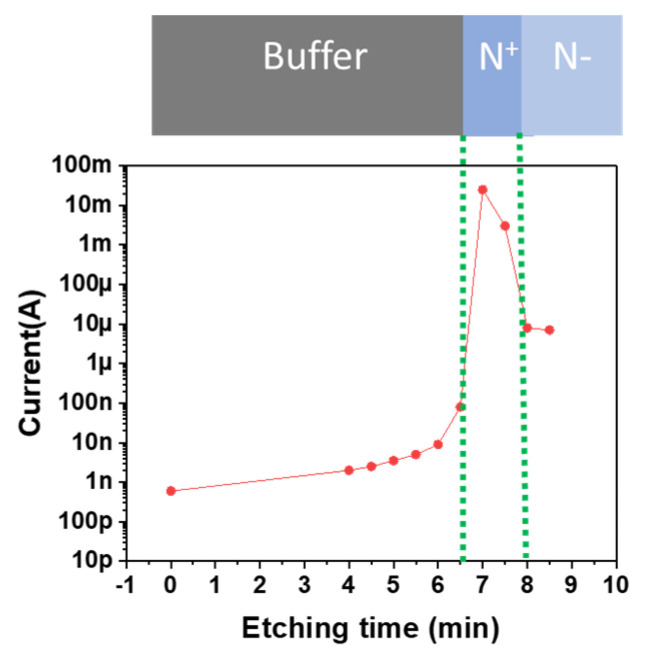
Current versus etching time curves to control the etch depth.

To avoid robustness issues of the membranes, the vertical GaN fabrication process started with the bonding of the front side on a Silicon substrate by thermal compression of gold at 300 °C (Figure 2a). The metal used for bonding was Ti (100 nm)/Au (200 nm), deposited by evaporation on both the Silicon substrate and the PIN diode frontside. The backside process began with thinning the Si substrate from 1 mm down to 150 µm, followed by the deposition and patterning of a thick resist layer using standard lithography. This resist layer served as a mask during the local removal of Si using deep reactive-ion etching. The buffer layer was subsequently removed using a Cl2 ICP (inductively coupled plasma)-based recipe. Due to the membrane’s critical etching depth, etching control is not trivial. Therefore, the etching time was calibrated by monitoring the electrical conductivity. Direct I(V) measurements on the surface (without metals) at 30 V resulted in the following current level for each layer: nA range for the buffer layers, mA range for the n+ layer, and µA range for the n- layer (Figure 3).

**Figure 4:**
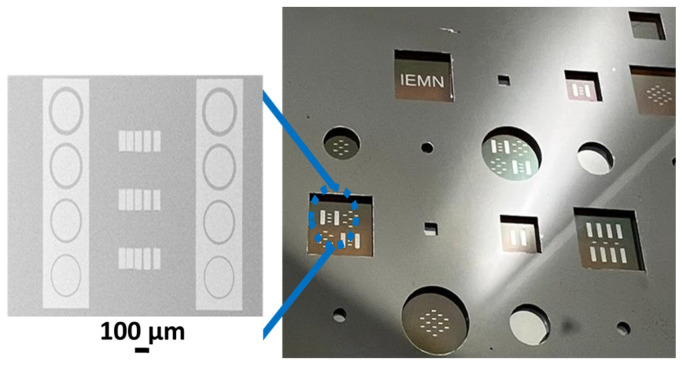
SEM and optical images of vertical GaN membranes with TLM patterns after metallization and lift-off. The rectangular membranes width are 1 mm, 3 mm, and 5 mm. The circular membrane diameters are 1 mm, 3 mm, and 5 mm.

Standard photolithography based on a hard mask to pattern the TLM through the resist was not feasible due to the large gap between the GaN membrane surface and the optical exposure level. Therefore, direct laser writing was applied to achieve the TLM patterns. The vertical device processing concluded with the deposition of a Ti (25 nm)/Al (100 nm) contact layer using evaporation and subsequent lift-off on the backside (Figure 4). The aforementioned recipe was used to generate several samples from the same wafer avoiding any epi-variation. The various samples were used to define the design of the experiment prior to the deposition of the contact layer as follows:

- Control samples B1 and B2 were metalized without any treatment, with a delay of 10 min and 24 h after buffer etching, respectively.
- To investigate the time dependence, samples C1, C2, C3, and C4 were treated with HCl (37%) at 70 °C for 1 min, 2 min, 3 min, and 5 min prior to metallization, respectively.
- To investigate the temperature dependence, samples D1, D2, and D3 were treated with HCl (37%) for 1 min at room temperature, 55 °C, and 70 °C prior to metallization, respectively.
- An additional sample, referred to as sample A, was prepared with a pseudo-vertical structure. This configuration involved contacting the n+ layer from the front side on the Ga-face after ICP mesa etching (Figure 2b) to compare the Ti/Al ohmic contact quality with the N-face from the same GaN layer.

Transmission line measurements (TLMs) were performed to assess the electrical quality of the ohmic contacts in all cases as described in [24,25]. Rectangular 50 × 100 µm^2^ TLM pads were used with 5 µm, 10 µm, 15 µm and 20 µm contact spacing (Figure 4) to extract the specific contact resistance. I(V) measurements within the range of [−10 V; 10 V] between 10 µm contact spacing are used to compare the contact quality of the various applied treatments. KEITHLEY 2612B enabling both 2 probes and 4 probes measurements was employed for the extraction of I(V) measurements and specific contact resistance extraction, respectively. 

In addition to electrical characterization, the samples were also structurally characterized using XPS and SEM. XPS analysis was performed right after the HCl treatment using a Sigma Probe instrument of Thermo Fisher Scientific Inc. with a base pressure of approximately 10^−^^9^ mbar. A monochromatic Al Kα X-ray source was used as the excitation source. The spectra were calibrated by setting the N-Ga component in the N1s spectra to 397.7 eV [26,27,28,29]. Next, the O-Ga to Ga ratio in the Ga3d spectra was determined by fitting the spectra using CasaXPS (Casa Software Ltd. 2.3.26). The error on the atomic concentrations obtained via CasaXPS is typically estimated to be roughly 10% of the reported value. After XPS, SEM images were obtained from the samples using an FEI Quanta 200F instrument. Cross-sectional images were obtained after cleaving the samples.

## 3. Results

**Figure 5:**
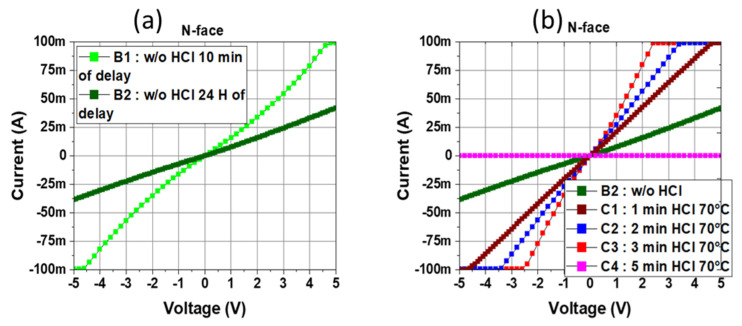
(**a**) TLM measurements of the N-face ohmic contact without treatment after various time durations between buffer etching and metal deposition. (**b**) N-face TLM measurements comparison of different HCl treatment times (0–5 min) at 70 °C.

Figure 5a shows TLM measurements on the N-face for a 10 min delay between the buffer etching and the deposition of metal (sample B1). The IV characteristics reveal a poor linear behavior, although a rather high current of 100 mA at 4.5 V is observed resulting in 2.4 × 10^−4^ Ω·cm^2^-specific contact resistance. When increasing the delay to 24 h (sample B2), the measured current drops to 40 mA at 5 V due to much higher specific contact resistance >10^−3^ Ω·cm^2^. These results suggest that an oxidation phenomenon occurs during the delay time. An HCl (37%)-based wet treatment was developed with a temperature initially fixed at 70 °C in agreement with reported results in [30] demonstrating the need for high temperatures to efficiently remove the native oxide.

**Figure 6:**
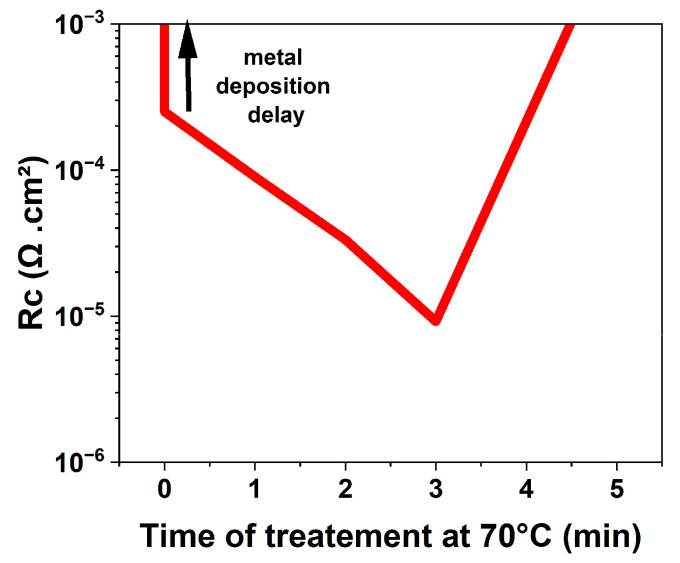
Rc versus time of treatment at 70 °C.

Figure 5b shows the time dependence of the HCl wet treatment at 70 °C on the ohmic-specific contact resistance. Up to 3 min time duration (samples C1, C2, and C3), the HCl treatment significantly reduces the specific contact resistance down to 9 × 10^−6^ Ω·cm^2^ without additional annealing (see Figure 6), making this approach fully compatible with any frontside post-process. On the other hand, when further increasing the treatment time up to 5 min and above (sample C4), the quality of the ohmic contacts severely deteriorates, and the extracted current drops dramatically, indicating a surface degradation due to prolonged treatment.

**Figure 7:**
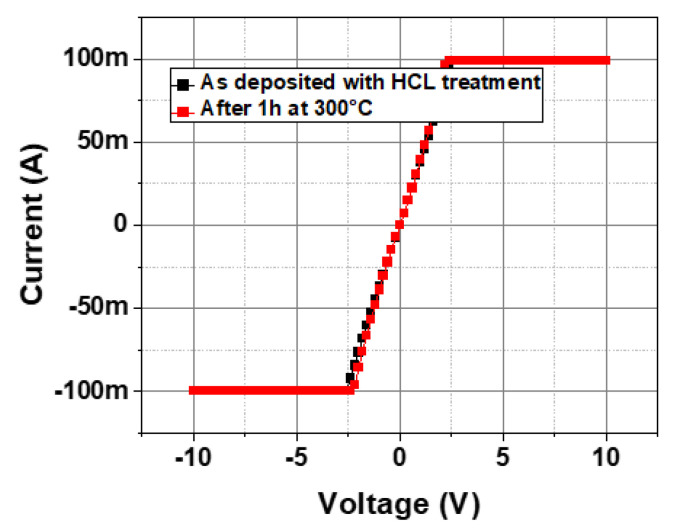
TLM measurements of the N-face ohmic contact with HCl pretreatment before and after thermal stress for 1 h at 300 °C.

An additional test was performed in order to gain some initial insights into the thermal stability of the ohmic contacts. Sample C3 (using the optimum conditions of 3 min HCl treatment at 70 °C) was introduced in an oven in a rich N2 environment at 300 °C for 1 h. Figure 7 shows the electrical current–voltage TLM measurements before and after 1 h thermal stress at 300 °C. The identical IV curves clearly indicate that the ohmic contacts are stable with no short-term impact of the temperature on the applied wet pretreatment.

**Figure 8:**
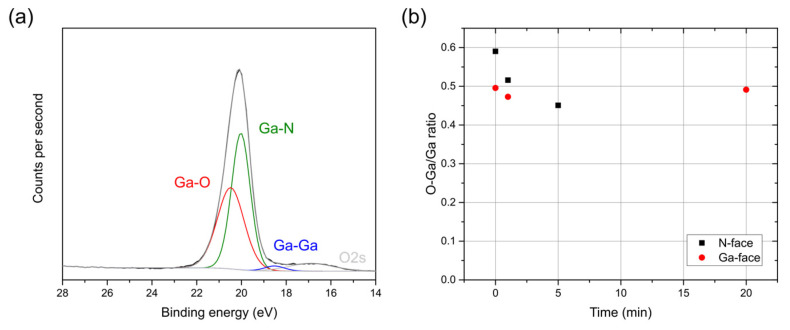
(**a**) Ga3d spectra of the 5 min HCl-treated N-face sample demonstrating how the spectra are fitted. (**b**) O-Ga/Ga ratio in the Ga3d spectra as measured by XPS as a function of HCl treatment time at 70 °C for N-face (squares) and Ga-face (circles).

To observe the chemical modification of the GaN surface with the HCl treatment, various N-face and Ga-face samples were treated with HCl at 70 °C for time durations ranging between 1 and 20 min and immediately measured by XPS. The oxidation state of the surface can be determined by deconvoluting the Ga3d spectra into three peaks: Ga-N, Ga-O, and Ga-Ga at a binding energy of 20.0–20.1 eV, 20.5–21.0 eV, and 18.5–19.1 eV, respectively [26,27,29]. As an example, the deconvolution of the Ga3d spectrum of the 5 min HCl-treated N-face sample can be found in Figure 8a. Using these fits, the O-Ga-to-Ga ratio is then calculated and plotted as a function of HCl treatment time at 70 °C in Figure 8b. The data show that the untreated N-face is oxidized to a greater extent than the untreated Ga-face. In addition, the HCl treatment is observed to reduce the N-face upon treatment time, while even after a treatment time of 20 min, the Ga-face remains roughly unaffected. These findings correspond well to the electrical results, showing no impact of the HCl treatment on the Ga-face, while an initial increase in contact quality is found upon removal of the Ga-O surface species for the N-face. The latter is also in agreement with the literature, reporting that the removal of native oxide from the GaN surface is crucial to obtaining low-resistance ohmic contacts [19,31]. 

**Figure 9:**
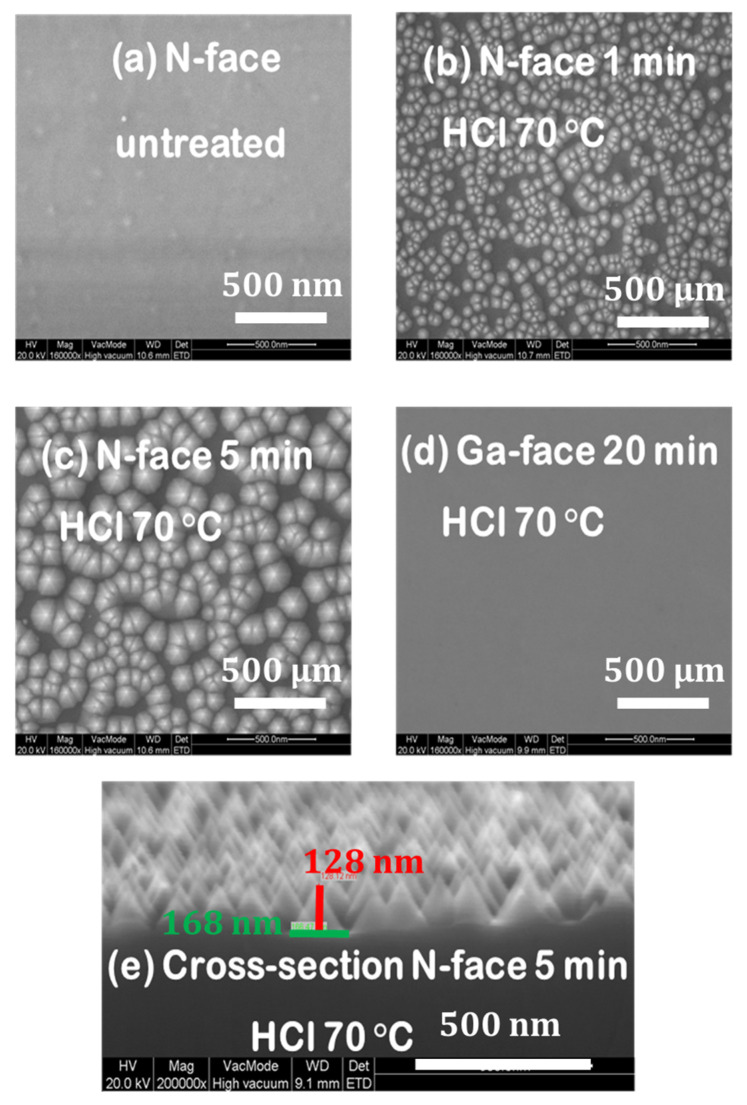
SEM images of the HCl-treated GaN surfaces.

SEM images display that the HCl treatment not only reduces the N-face but can also be associated with the formation of hexagonal pyramid-shaped features at the surface, as displayed in Figure 9. While the Ga-face again remains unaffected even after the 20 min HCl treatment at 70 °C (Figure 9d), the features are observed to enlarge upon treatment time for the N-face (Figure 9a–c). After the 5 min HCl treatment, the height and width of the features are estimated to be approximately 128 nm and 166 nm, respectively (Figure 9e). Similarly, [32] report the formation of sixfold nanopyramids on N-face GaN after etching for 45 min in 2 M KOH at 90 °C, whereas the Ga-face is observed to remain smooth. Their observations demonstrate that the hexagonal pyramid shape originates from the preferential etching of crystalline planes. Also, [33,34] report the formation of pyramid shapes that are observed to increase over time after etching in heated KOH or H3PO4 solutions. 

The formation of these features is accompanied by an increase in the roughness of the surface. Hypothetically, this increase in roughness might explain why the 5 min HCl-treated sample behaves poorly in comparison to the 3 min HCl-treated sample as observed by TLM. This would suggest that an optimal HCl treatment time exists on N-face GaN, showing the perfect trade-off between native oxide removal and surface roughness increase to obtain the lowest specific contact resistance.

**Figure 10:**
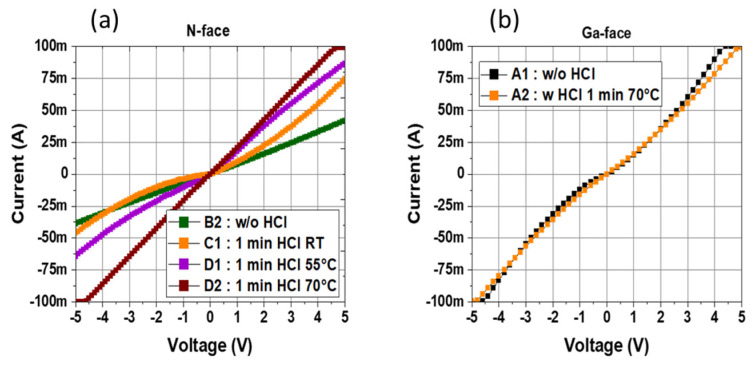
(**a**) N-face TLM measurements for samples treated with HCl treatment at different temperatures (RT, 55 °C, and 70 °C) (**b**) Ga-face TLM measurements comparison with and without HCL treatment.

Figure 10a compares TLM measurements of different samples treated with HCl for 1 min at various temperatures to study the impact of treatment temperature on the ohmic contact quality. Sample B2 is the control sample without any treatment. It can be observed that room temperature (RT) treatment (sample D1) improves the TLM characteristics with a higher current. This is also the case for the 55 °C treatment (sample D2), which still delivers high specific contact resistance >10^−3^ Ω.cm^2^. The most effective treatment temperature was found to be 70 °C (sample C1), resulting in a significantly improved current level with an associated specific contact resistance of 7–8 10^−5^ Ω.cm^2^. It is important to note that additional samples were treated with longer treatment times at RT and 55 °C, but the ohmic-specific contact resistance never reached the ones achieved at 70 °C.

For a fair benchmark, specific contact resistance on the Ga-face n+ GaN layer was measured with (sample A1) and without HCl treatment at 70 °C (sample A2) (Figure 10b). Despite the absence of pretreatment, Ga-face-specific contact resistance is comparable to the N-face in sample B1 immediately between buffer etching and metallization. Furthermore, no impact of HCl wet treatment at 70 °C was observed on the Ga-face ohmic contacts that generally require extra annealing to further reduce the specific contact resistance. These results confirm that the properties of N-face are significantly different from those of the Ga-face GaN layer, suggesting that the native oxide of the N-face GaN layer is the major factor influencing the ohmic contact quality but can be mitigated using a proper wet pretreatment without any annealing.

Table 1 summarizes the sample while indicating the polarity, the treatment, and the extracted specific contact resistance.

## 4. Conclusions

In this work, N-face backside ohmic contacts in fully vertical GaN-on-Silicon PIN diodes are assessed. Direct laser writing lithography enabled TLMs inside the membrane to be patterned, as standard photolithography could not be used in this frame. TLM measurements from as-deposited metal stacks showed high specific contact resistance, which degrades over time between membrane opening and metal deposition. This suggests a gradual oxidation of the N-face GaN. Therefore, an HCl treatment was developed and found to be effective in reducing specific contact resistance and improving the maximum current. The optimum HCl treatment was found to be 3 min at 70 °C while the contacts were severely degraded for a longer time. It can be pointed out that short-term thermal stability up to 300 °C shows no degradation of the optimized ohmic contacts. XPS analysis revealed a clear reduction in Ga-oxide with the HCl treatment. Moreover, SEM images showed the formation of pyramids, especially for prolonged HCl treatment, explaining ohmic contact drastic degradation for a longer time. Finally, it has also been shown that the treatment temperature of 70 °C is critical as far less improvement occurs when the temperature is reduced. This simple approach can be used for fabricating any type of fully vertical GaN-on-Silicon devices as no extra annealing is required to achieve negligible backside-specific contact resistance and, thus, is fully compatible with frontside processing. 

## Figures and Tables

**Table 1:** Summary of the samples, polarity, and the specific contact resistance.

Sample	Polarity	Treatment	Specific Contact Resistance (Ω·cm^2^)
A1	Ga face	No treatment	10^−4^
A2	Ga face	No treatment	10^−4^
B1 (no delay)	N face	No treatment	2.4 × 10^−4^
B2 (24 H delay)	N face	N treatment	High
C1	N face	1 min 70 °C of HCL	7–8 × 10^−5^
C2	N face	2 min 70 °C of HCL	3.3 × 10^−6^
C3	N face	3 min 70 °C of HCL	9 × 10^−6^
C4	N face	5 min 70 °C of HCL	High
D1	N face	1 min RT of HCL	High
D2	N face	1 min 55 °C of HCL	High

## Data Availability

The data presented in this work are available within the article.
